# Contemporary national trends in the management of admissions for pulmonary embolism

**DOI:** 10.3389/fcvm.2026.1757697

**Published:** 2026-03-13

**Authors:** Connor G. Oltman, Kellie Cao, Maaz Imam, Riyaz Bashir, Suhail Dohad, Eric A. Secemsky, Issam D. Moussa

**Affiliations:** 1 Carle Illinois College of Medicine, University of Illinois Urbana-Champaign, Urbana, IL, United States; 2 Division of Cardiovascular Diseases, Lewis Katz School of Medicine, Temple University, Philadelphia, PA, United States; 3 Cedars Sinai Medical Center, Beverly Hills, CA, United States; 4 Division of Cardiovascular Medicine, Beth Israel Deaconess Medical Center, Harvard Medical School, Boston, MA, United States; 5 Richard A. and Susan F. Smith Center for Outcomes Research in Cardiology, Boston, MA, United States; 6 Heart and Vascular Institute, Carle Health, Urbana, IL, United States

**Keywords:** anticoagulation, mechanical thrombectomy, mechanical ventilation, pulmonary embolism, reperfusion therapies

## Abstract

**Introduction:**

Over the past decade, the inpatient management of pulmonary embolism has undergone a paradigm shift in response to new evidence and the adoption of catheter-directed reperfusion therapies. However, real-world practice patterns remain poorly characterized.

**Methods:**

This study used Epic's Cosmos database to analyze admissions for pulmonary embolism in the United States between January 1, 2016, and December 31, 2024. Adult inpatient admissions for pulmonary embolism were identified by International Classification of Diseases, Tenth Revision codes documented in the *Admit to Inpatient* order. Admissions were stratified by severity using established high-risk criteria to allow for a risk-based comparison of treatment strategies. High-risk criteria included the presence of cardiogenic shock, cardiac arrest, the use of vasopressors, dobutamine, extracorporeal membrane oxygenation, or mechanical ventilation at any point during the admission; the remaining admissions were classified as non-high-risk. Trends in patient characteristics, anticoagulation strategies, reperfusion therapies, and cardiopulmonary support were analyzed across the study period.

**Results:**

This study identified a total of 267,094 hospital admissions for pulmonary embolism (mean [SD] age, 63 [17] years; 51.4% female; 71.1% White) between 2016 and 2024. Of these admissions, 5.5% met one or more high-risk criteria, increasing from 4.3% in 2016 to 5.8% in 2024 (*p* < 0.001). The proportion of patients receiving unfractionated heparin alone increased across non-high-risk (33.2% to 63.0%) and high-risk cases (53.7% to 66.3%). Among non-high-risk admissions, the utilization of reperfusion therapies nearly doubled (5.2% to 10.3%, *p* = 0.002), primarily driven by a rise in the use of catheter-directed embolectomy. In high-risk admissions, the overall use of reperfusion therapies remained stable (27%–34%, *p* = 0.135), while catheter-directed embolectomy emerged as the predominant modality. For hemodynamic support of high-risk admissions, vasopressor/dobutamine utilization increased (53.3% to 72.2%) as mechanical ventilation use declined (54.7% to 32.4%).

**Conclusions:**

These findings help contextualize the extent to which novel therapies and evolving practice patterns have been integrated into real-world care in the United States. Catheter-directed embolectomy has become the dominant reperfusion strategy for pulmonary embolism, reflecting a major shift in practice.

## 1 Introduction

Pulmonary embolism (PE) remains a major contributor to cardiovascular morbidity and mortality (1, 2), with significant public health (3) and economic implications (4). Despite advances in prevention and treatment, PE contributes to at least 50,000 deaths annually in the United States (U.S.) (5). The growing prevalence of key risk factors, including obesity and cancer, along with an aging population, has driven a marked rise in PE incidence (6–8). Concurrently, improvements in diagnostic imaging have increased the detection of PE (9, 10). Between 1993 and 2012, annual hospitalizations for PE more than tripled, increasing from approximately 60,000 cases (23 per 100,000 population) to over 202,000 cases (65 per 100,000 population) (8). More recently, PE diagnoses in emergency departments have nearly quadrupled, rising from 0.1% of all encounters in 2010–2012 to 0.35% in 2021 (11, 12).

As the clinical burden of PE has steadily increased, its management has undergone a paradigm shift in response to new therapies and evolving evidence. Clinical interest in catheter-directed therapies has grown over the past two decades (13), leading to new treatment options for select patients (14). However, their role within guideline-directed therapy is still evolving, and their real-world adoption is not well characterized. Similarly, while direct oral anticoagulants (DOACs) have become the preferred long-term anticoagulation strategy (6), patterns of inpatient anticoagulation remain underexplored. Finally, emerging data suggests that urgent intubation in critically ill patients may worsen outcomes (15, 16), challenging conventional management approaches. Despite these changes to hospital-based PE management, real-world practice patterns across the spectrum of disease severity have not been systematically characterized, particularly on a national scale.

This study evaluated trends in the inpatient management of PE in the U.S. from 2016 to 2024, capturing shifts in practice across high-risk and non-high-risk presentations. Spanning nearly a decade of real-world data, this analysis provides critical insight into evolving treatment patterns that may inform future guidelines and improve patient care.

## 2 Methods

### 2.1 Data source

Data used in this study came from Epic Cosmos (17), a dataset created in collaboration with a community of Epic health systems representing more than 289 million patient records from over 1,626 hospitals and 37,700 clinics from all 50 states, the District of Columbia, Lebanon, and Saudi Arabia. Cosmos provides a Health Insurance Portability and Accountability Act-defined limited dataset, consolidating patient records across multiple healthcare organizations to create unified, comprehensive profiles (18). The database includes patient demographics, medications, procedures, and diagnoses, retrieved using International Classification of Diseases, Tenth Revision (ICD-10) and Current Procedural Terminology (CPT) codes. This study used deidentified aggregate data and was not classified as human subject research; therefore, approval from the Carle Institutional Review Board was not required. The findings of this study are reported in accordance with the Strengthening the Reporting of Observational Studies in Epidemiology (STROBE) guidelines for cohort studies (19).

### 2.2 Study population

**Figure 1:**
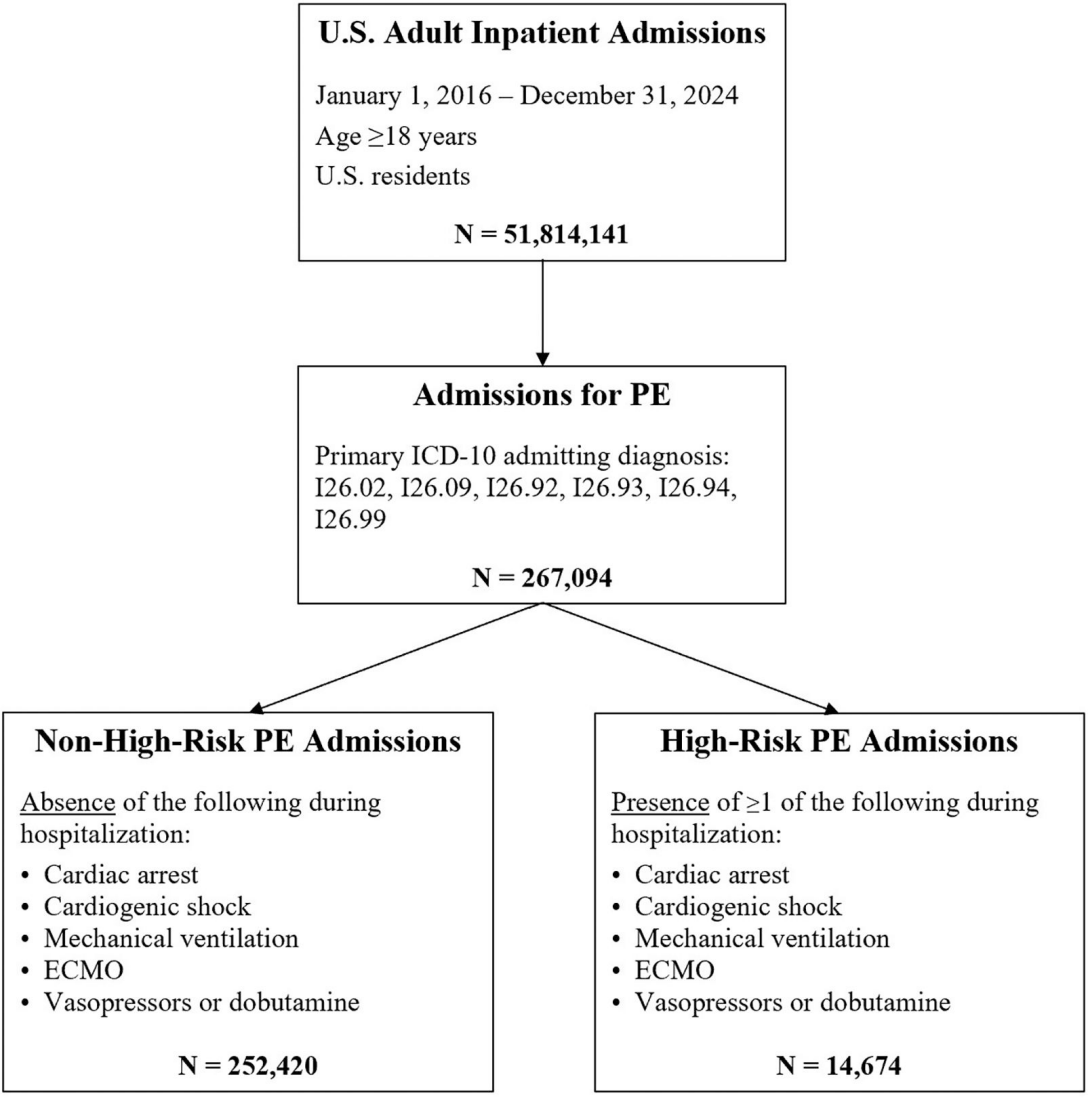
Flow diagram of study cohort. Flow diagram demonstrating identification of U.S. adult inpatient admissions for PE between 2016 and 2024 and subsequent stratification into non-high-risk and high-risk groups. ECMO, extracorporeal membrane oxygenation; ICD-10, international classification of diseases, tenth revision; PE, pulmonary embolism.

A cohort of U.S. hospital admissions for PE was assembled, including adults aged 18 years or older admitted between January 1, 2016, and December 31, 2024 (Figure 1). These admissions were identified by ICD-10 codes for venous thromboembolic PE (I26.02, I26.09, I26.92, I26.93, I26.94, I26.99) documented as the primary admitting diagnosis in the *Admit to Inpatient* order at the time of hospitalization or during admission registration. A prior study demonstrated that these codes have a sensitivity >91% and a specificity >99% for identifying PE diagnoses in the emergency department (20). Admissions coded for septic PE were excluded due to its distinct pathophysiology from venous thromboembolic PE. Each qualifying admission was counted independently; patients with multiple PE admissions during the study period were included for each eligible hospitalization. Inter-facility transfers were treated as separate hospitalizations if a new qualifying admission with a primary diagnosis of PE was generated at the receiving institution.

Admissions were then stratified by PE severity using high-risk criteria from prior studies (21, 22). Because granular hemodynamic parameters (e.g., sustained hypotension or objective right ventricular dysfunction) are not reliably captured in the Cosmos database, high-risk PE was operationally defined using diagnostic codes for hemodynamic compromise and the receipt of advanced supportive therapies. Admissions were classified as high-risk if the patient experienced cardiac arrest or cardiogenic shock at any point during hospitalization. In addition, high-risk classification incorporated downstream therapeutic interventions occurring during hospitalization, including mechanical ventilation, extracorporeal membrane oxygenation (ECMO), and vasopressor or dobutamine use. The remaining admissions were categorized as non-high-risk. To assess the influence of defining high-risk PE using downstream interventions, we performed a sensitivity analysis defining high-risk PE using diagnosis-based criteria only (cardiac arrest and/or cardiogenic shock) and re-evaluated key temporal trends. The ICD-10 and CPT codes used to define these criteria are detailed in Supplementary Table S1. The proportion of admissions that met one or more of these high-risk criteria was assessed annually from 2016 to 2024.

### 2.3 Patient characteristics

Patient characteristics (age, sex, race/ethnicity, and comorbidities), hospital length of stay, and clinical presentation were compared across non-high-risk and high-risk admissions for 2016 and 2024. Race and ethnicity were determined using self-reported data from the electronic health record, mapped to standard Centers for Disease Control and Prevention categories within the Cosmos database. Non-Hispanic White individuals were classified as “White”, with a similar approach applied to other racial and ethnic categories. Admissions with missing race/ethnicity data were excluded from analyses involving those variables. Comorbidities, including anemia, cancer, coronary artery disease, diabetes mellitus, dyslipidemia, heart failure, and hypertension, were identified using ICD-10 codes (Supplementary Table S2).

### 2.4 Management

Inpatient management trends, including anticoagulation (parenteral and oral) and reperfusion therapies were analyzed annually from 2016 to 2024 across non-high-risk and high-risk admissions. Anticoagulation was assessed for parenteral agents, including unfractionated heparin (UFH), low-molecular-weight heparin (LMWH), and fondaparinux, as well as oral agents, including apixaban, dabigatran, rivaroxaban, and warfarin, with overall use defined as administration of at least one agent during the admission. Eligible medications included those administered by clinicians in-house and documented within Epic's *Medication Administration Record*. Reperfusion therapies included catheter-directed embolectomy (CDE), catheter-directed thrombolysis, surgical embolectomy, and systemic thrombolysis, with overall utilization defined as receipt of any intervention during hospitalization. Cardiopulmonary support (ventilation, ECMO, and vasopressors/dobutamine) was assessed among high-risk admissions. Reperfusion procedures were identified using ICD-10 codes (Supplementary Table S3).

### 2.5 Statistical analysis

Patient characteristics and management trends were analyzed separately for non-high-risk and high-risk PE. Continuous variables are reported as means with standard deviations and were compared using the Student's *t*-test. Categorical variables are presented as counts with percentages and were compared using the *χ*^2^ test. Wilson score 95% confidence intervals were calculated for low-frequency outcomes, including surgical embolectomy and ECMO, and are reported in Supplementary Table S4. Temporal trends in the proportion of high-risk PE, overall reperfusion therapy utilization, and overall anticoagulant utilization were evaluated using the Cochran-Armitage test. All *p*-values are two-sided, with *p* < 0.05 considered statistically significant. Statistical analyses were conducted using Python (version 3.12.8, Python Software Foundation).

## 3 Results

### 3.1 Patient characteristics

**Figure 2:**
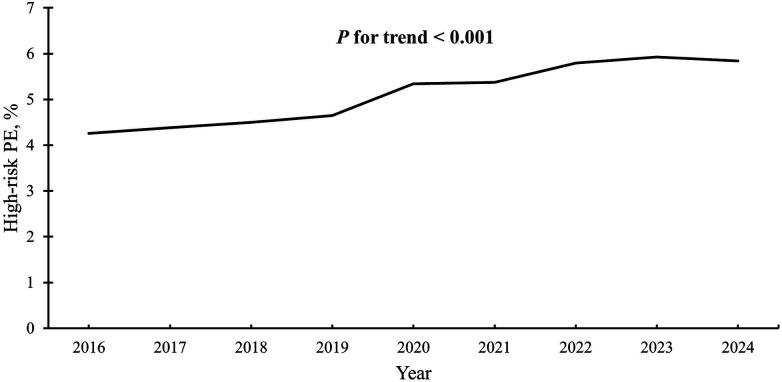
Trends in the proportion of admissions for high-risk pulmonary embolism. High-risk PE was defined as admissions complicated by cardiogenic shock or cardiac arrest, as well as those requiring vasopressors, dobutamine, mechanical ventilation, or ECMO. The proportion of high-risk PE was calculated as the number of cases meeting these criteria divided by the total number of admissions for PE. The proportion of high-risk PE significantly increased over time (*P* for trend < 0.001). ECMO, extracorporeal membrane oxygenation; PE, pulmonary embolism.

This study identified 267,094 hospital admissions for PE between 2016 and 2024 (mean [SD] age, 63 (17) years; 51.4% female; 71.1% White). Among these, 14,674 admissions (5.5%) met at least one high-risk criterion. The proportion of high-risk admissions increased from roughly 4.3% in 2016 to 5.8% in 2024, reflecting a 37.1% relative increase over the study period (Figure 2). In a sensitivity analysis defining high-risk PE using diagnosis-based criteria only (cardiac arrest and/or cardiogenic shock), the proportion of high-risk PE was lower, but temporal trends were directionally consistent with the primary analysis (Supplementary Figure S1).

While some patient characteristics remained stable, others evolved among non-high-risk and high-risk PE admissions (Table 1). The proportion of female patients, the racial/ethnic distribution of patients, hospital length of stay, intensive care unit utilization, and weekend admissions remained relatively constant throughout the study period. From 2016 to 2024, mean admission age rose from 61 to 65 years in non-high-risk PE and 61 to 67 in high-risk PE (both *p* < 0.001). Additionally, nearly all comorbidities became more prevalent over time, and ambulance use was higher in 2024 compared to 2016.

**Table 1:** Patient characteristics, comorbidities, and clinical presentation of admissions for non-high-risk and high-risk pulmonary embolism in 2016 and 2024.

Patient characteristics	Non-high-risk PE			High-risk PE		
	2016 (*n* = 6,208)	2024 (*n* = 61,985)	*P* value	2016 (*n* = 276)	2024 (*n* = 3,843)	*P* value
Age, y, mean (SD)	61 (17)	65 (16)	<0.001	61 (15)	67 (15)	<0.001
Female sex, *n* (%)	3,217 (51.8)	32,067 (51.7)	0.896	135 (48.9)	2,051 (53.4)	0.152
Hospital length of stay, days, mean (SD)	4.3 (3.7)	4.3 (3.9)	1.000	9.1 (7.6)	8.2 (7.3)	0.900
Race and ethnicity, *n* (%)^a^						
American Indian or Alaska Native	22 (0.4)	337 (0.5)	0.049	≤10 (≤3.6)^b^	17 (0.4)	<0.001^c^
Asian or Pacific Islander	48 (0.8)	805 (1.3)	<0.001	≤10 (≤3.6)^b^	51 (1.3)	0.002^c^
Black or African American	1,230 (19.8)	13,428 (21.7)	<0.001	70 (25.4)	873 (22.7)	0.312
Hispanic	186 (3.0)	2,972 (4.8)	<0.001	≤10 (≤3.6)^b^	194 (5.0)	0.292^c^
White	4,647 (74.9)	42,798 (69.0)	<0.001	192 (69.6)	2,583 (67.2)	0.421
Comorbidities, *n* (%)						
Hypertension	3,762 (60.6)	42,307 (68.3)	<0.001	176 (63.8)	2,798 (72.8)	0.001
Diabetes	1,333 (21.5)	16,313 (26.3)	<0.001	77 (27.9)	1,267 (33.0)	0.080
Dyslipidemia	2,196 (35.4)	30,530 (49.3)	<0.001	97 (35.1)	2,003 (52.1)	<0.001
Heart Failure	798 (12.9)	12,918 (20.8)	<0.001	87 (31.5)	1,615 (42.0)	<0.001
Coronary Artery Disease	993 (16.0)	13,340 (21.5)	<0.001	62 (22.5)	1,108 (28.8)	0.023
Active Malignancy^d^	1,115 (18.0)	12,734 (20.5)	<0.001	57 (20.7)	916 (23.8)	0.241
Anemia	1,408 (22.7)	19,554 (31.5)	<0.001	119 (43.1)	2,149 (55.9)	<0.001
Presentation, *n* (%)						
ICU admission	1,361 (21.9)	13,537 (21.8)	0.878	249 (90.2)	3,304 (86.0)	0.048
Weekend admission	1,445 (23.3)	14,461 (23.3)	0.924	72 (26.1)	956 (24.9)	0.654
Ambulance use	1,631 (26.3)	23,272 (37.5)	<0.001	130 (47.1)	2,425 (63.1)	<0.001

PE, pulmonary embolism; ICU, intensive care unit.

a Admissions with missing race/ethnicity data were excluded from analyses involving those variables.

b Queries yielding ≤10 patients are masked in the Cosmos database. For percentage calculations, a value of 10 was used.

c *P* values were calculated assuming a count of 10 patients when ≤10 patients were returned.

d Active malignancy was defined as a cancer diagnosis documented during the admission encounter.

### 3.2 Anticoagulation

**Figure 3:**
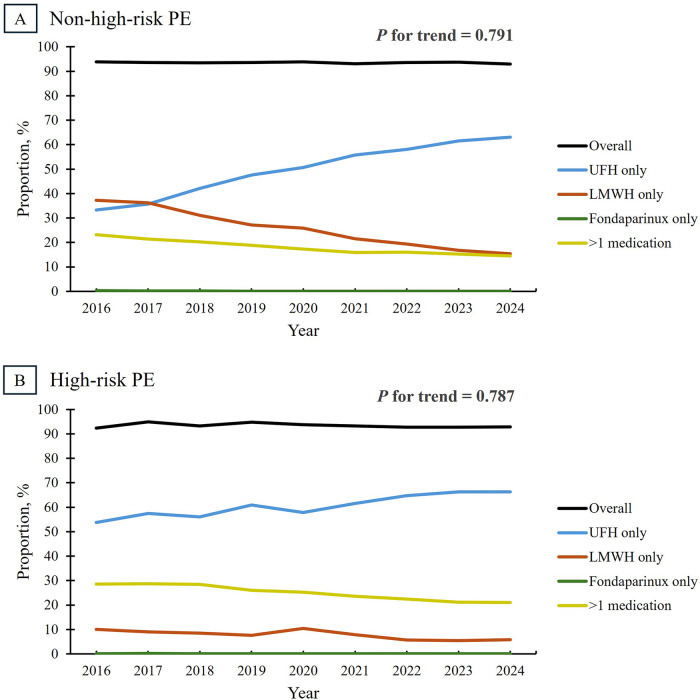
Trends in parenteral anticoagulation use among patients admitted for pulmonary embolism, stratified by risk category. Overall parenteral anticoagulation use, defined as receipt of UFH, LMWH, or fondaparinux during the hospitalization, remained stable among admissions for non-high-risk PE (Panel **A**) and high-risk PE (Panel **B**) at roughly 93% (*P* for trend = 0.791 and 0.787, respectively). However, a shift in anticoagulant preference was observed, marked by an increased use of UFH and a corresponding decline in LMWH utilization, particularly among admissions for non-high-risk PE. LMWH, low-molecular-weight heparin; PE, pulmonary embolism; UFH, unfractionated heparin.

Overall use of parenteral anticoagulation remained stable at approximately 93% among non-high-risk and high-risk admissions, with no significant change over time (*P* for trend = 0.791 and 0.787, respectively) (Figure 3). However, there was a substantial shift in the preferred parenteral anticoagulant. In non-high-risk admissions, the proportion of patients treated exclusively with UFH increased from 33.2% in 2016 to 63.0% in 2024, while exclusive use of LMWH declined from 37.2% to 15.4%. Additionally, the use of multiple parenteral agents declined from 23.2% to 14.5%. Among high-risk admissions, the use of UFH alone rose from 53.7% to 66.3%, the use of LMWH declined from 10.0% to 5.8%, and the use of multiple agents decreased from 28.5% to 21.0%. Across both groups, fondaparinux was rarely used alone (<0.5% of admissions).

**Figure 4:**
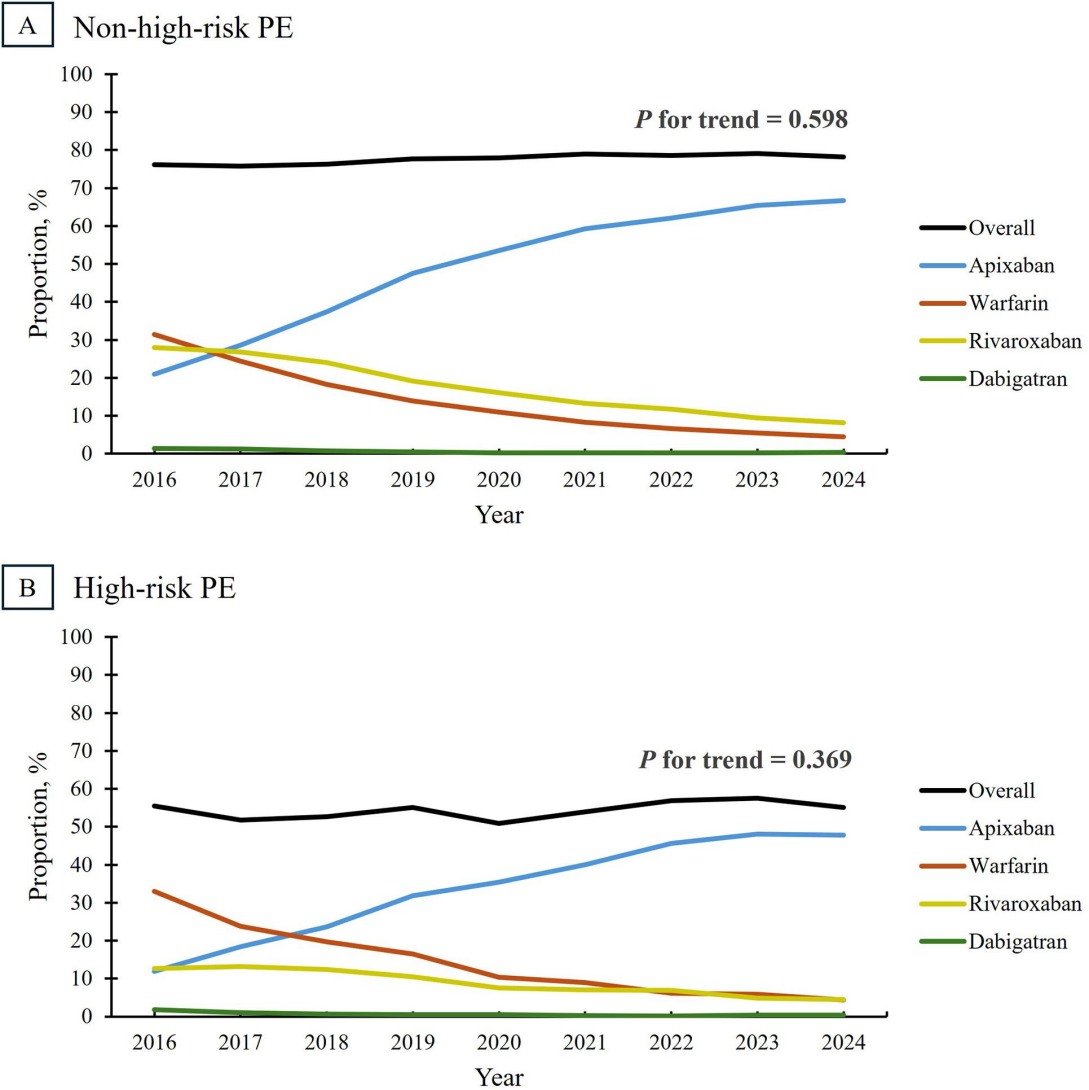
Trends in oral anticoagulation use among patients admitted for pulmonary embolism, stratified by risk category. Overall oral anticoagulation use, defined as receipt of one or more medications during the hospitalization, remained stable in non-high-risk PE (Panel **A**) and high-risk PE (Panel **B**) between 2016 and 2024 (*P* for trend = 0.598 and 0.369, respectively). Roughly 80% of patients with non-high-risk PE received at least one oral anticoagulant, compared to only 55% of those with high-risk PE. PE, pulmonary embolism.

Overall use of oral anticoagulants remained stable in both risk groups, with roughly 80% of non-high-risk patients (*p* = 0.598) and 55% of high-risk patients (*p* = 0.369) receiving at least one medication. By 2024, apixaban was the predominant oral anticoagulant used in non-high-risk (66.7%) and high-risk PE (47.8%), as seen in Figure 4. Apixaban became the preferred agent by 2017 in the non-high-risk group and by 2018 in the high-risk group, reflecting a slower adoption rate in the latter. Meanwhile, warfarin, the favored anticoagulant in both groups in 2016, declined in use, ranking as the second least preferred option by 2024. For both parenteral and oral anticoagulation, findings were qualitatively similar in sensitivity analyses restricted to diagnosis-defined high-risk PE (Supplementary Figures S2, S3).

### 3.3 Reperfusion therapies

**Figure 5:**
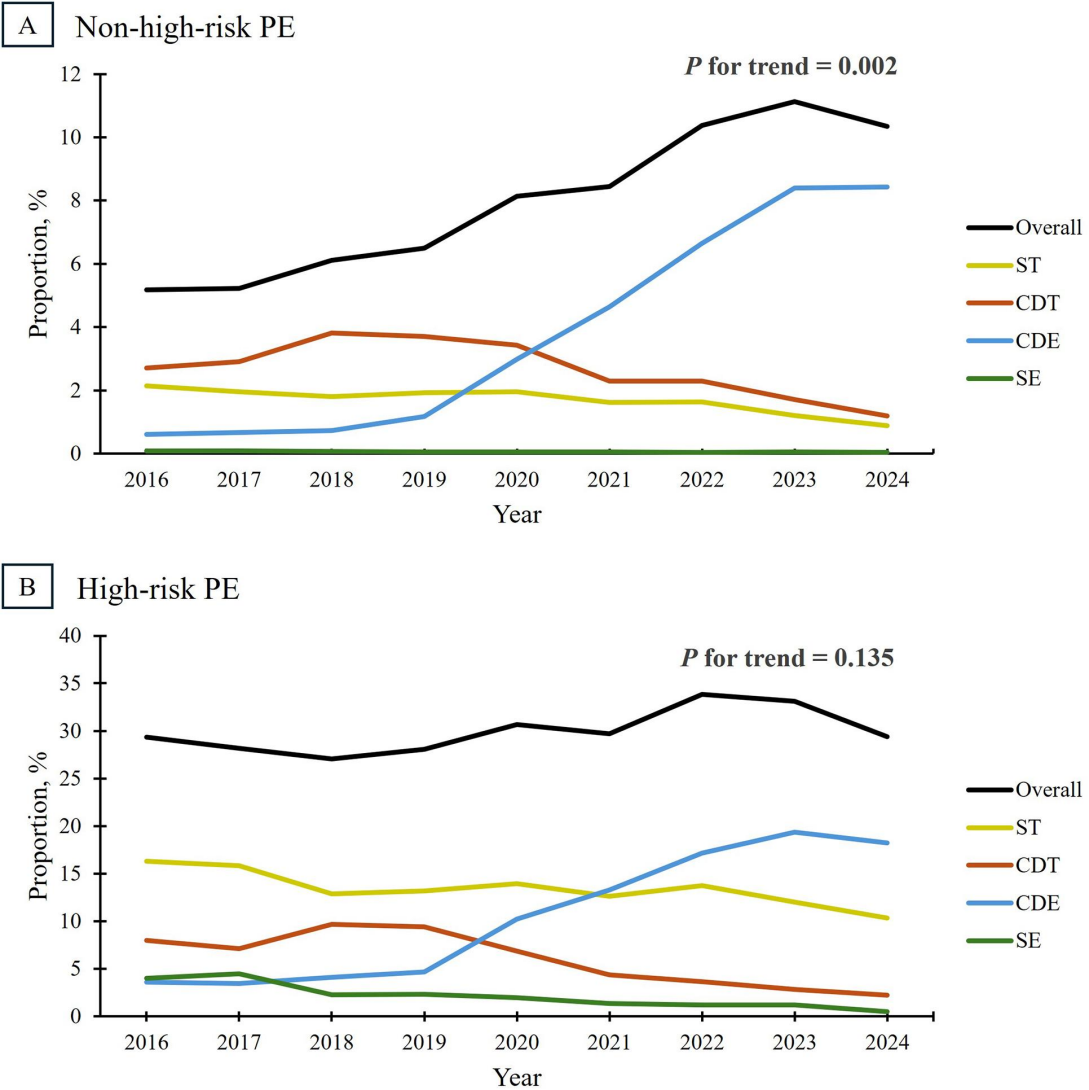
Trends in reperfusion therapy utilization among patients admitted for pulmonary embolism, stratified by risk category. In the non-high-risk group (Panel **A**), the overall use of reperfusion therapies significantly increased from 5.2% in 2016 to 10.3% in 2024 (*P* for trend = 0.002). In high-risk PE (Panel **B**), overall reperfusion therapy use remained stable (*P* for trend = 0.135), where roughly 30% of patients received at least one intervention. In both groups, CDE utilization increased over time, coinciding with a decline in ST, CDT, and SE use. CDE, catheter-directed embolectomy; CDT, catheter-directed thrombolysis; PE, pulmonary embolism; SE, surgical embolectomy; ST, systemic thrombolysis.

In the non-high-risk group, overall reperfusion therapy utilization nearly doubled from 5.2% in 2016 to 10.3% in 2024 (*p* = 0.002) (Figure 5). This trend was primarily driven by a dramatic increase in the use of CDE, which grew from 0.6% in 2019 to 8.4% in 2024, a nearly fourteen-fold increase over nine years. Conversely, catheter-directed thrombolysis increased early in the study period (from 2.7% in 2016 to 3.8% in 2018) but declined thereafter as CDE became the preferred approach, reaching 1.2% in 2024. Additionally, the use of systemic thrombolysis steadily declined throughout the study period, from 2.1% in 2016 to 0.9% in 2024. Surgical embolectomy utilization remained consistently low (<0.1%) in this group.

Among admissions for high-risk PE, reperfusion therapies were more commonly used than in the non-high-risk group, but remained relatively stable at 29.3% in 2016 to 29.4% in 2024, with no significant trend observed (*p* = 0.135). Like the non-high-risk group, CDE use increased rapidly from 3.6% in 2016 to 18.2% in 2024, reflecting a five-fold increase over nine years. In contrast, catheter-directed thrombolysis utilization rose briefly (from 8.0% in 2016 to 9.7% in 2018) before declining to 2.3% in 2024. Systemic thrombolysis, catheter-directed thrombolysis, and surgical embolectomy were used less frequently in 2024 compared to 2016. Findings were qualitatively similar in sensitivity analyses restricted to diagnosis-defined high-risk PE (Supplementary Figure S4).

### 3.4 Hemodynamic support

**Figure 6:**
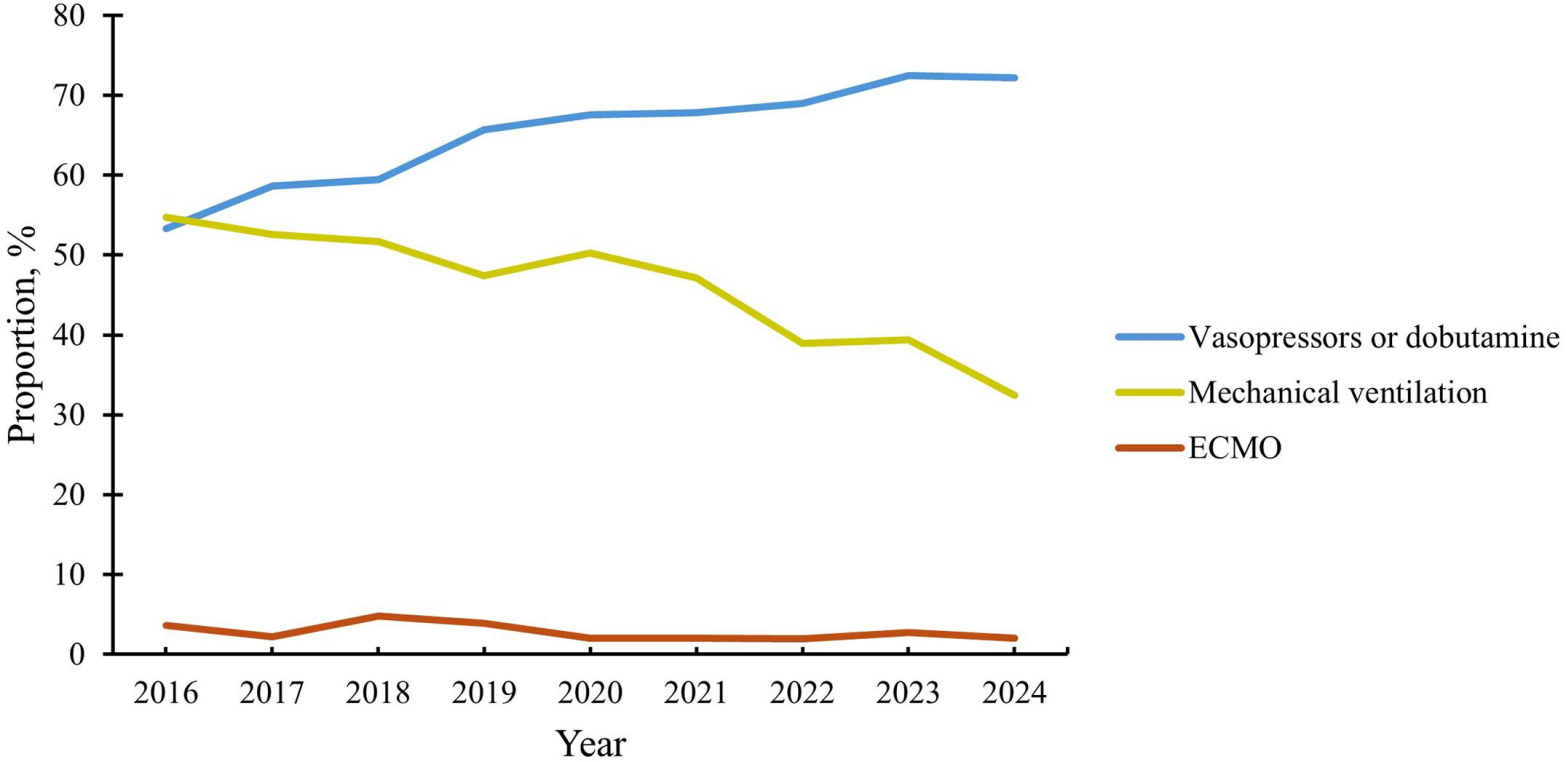
Trends in cardiopulmonary support among patients admitted for high-risk pulmonary embolism. This figure depicts trends in the use of vasopressors/dobutamine, mechanical ventilation, and ECMO among patients admitted for high-risk PE between 2016 and 2024. Over the study period, the use of vasopressors/dobutamine steadily increased, while utilization of mechanical ventilation declined, reflecting a shift toward strategies that prioritize hemodynamic stabilization while minimizing invasive respiratory support. ECMO, extracorporeal membrane oxygenation; PE, pulmonary embolism.

At the start of the study period, mechanical ventilation and vasopressors/dobutamine were each used in approximately 55% of high-risk PE admissions (Figure 6). By 2024, vasopressor/dobutamine use increased to 72.2%, while the use of mechanical ventilation declined to 32.4%. The use of ECMO remained low throughout the study period (<5% of high-risk admissions). Findings were qualitatively similar in sensitivity analyses restricted to diagnosis-defined high-risk PE (Supplementary Figure S5).

## 4 Discussion

### 4.1 Patient characteristics

This study is the first to comprehensively analyze contemporary trends in the inpatient management of PE across the U.S. Amid significant changes made to PE management over the past decade, these findings help contextualize the extent to which novel therapies and evolving practice patterns have been integrated into clinical practice.

The growing proportion of high-risk PE observed in this study aligns with prior research, which reported a rise from 2.0% in 1999 to 3.1% in 2014 (23). Several shifts may be driving this trend. By the end of the study period, the prevalence of cancer, hypertension, heart failure, and older age at admission had increased, all established risk factors for PE (24). As the population ages and the burden of comorbid conditions grows, the proportion of high-risk PE is likely to increase further. Additionally, changes in management patterns may have influenced the inpatient case mix. In one study, 11.8% of patients with confirmed PE were managed in the outpatient setting, with adoption of this approach increasing by 73% between 2000–2004 and 2005–2009 (25). As outpatient management of low-risk PE becomes more common, hospitalized cases may increasingly represent more severe clinical presentations.

### 4.2 Anticoagulation

While current guidelines recommend LMWH over UFH for parenteral anticoagulation in acute PE (6), recent studies have identified a growing shift towards UFH use in clinical practice (26). This study corroborates prior observations and offers new insight into a potential driver of this trend. Among a constellation of contributing factors, including institutional culture, therapeutic inertia, and misconceptions about anticoagulant pharmacology, the anticipated need for catheter-based intervention has emerged as a key influence guiding the selection of UFH (27). In this context, the sharp rise in CDE utilization, particularly among non-high-risk cases, who represent the majority of PE admissions, may be steering clinicians towards UFH.

A common misconception holds that anticoagulation must be withheld or reversed before catheter-directed therapies (27); in practice, anticoagulation is routinely continued during these procedures and can safely include LMWH (28, 29). Similarly, LMWH does not preclude the use of rescue thrombolysis, as demonstrated in trials such as TOPCOAT (30). While UFH remains appropriate in select scenarios, such as severe renal dysfunction, severe obesity, or imminent invasive surgical procedures, these circumstances are relatively uncommon (6). Dispelling this misconception is essential to align real-world practice with evidence-based recommendations. Without such efforts, the expanding role of catheter-directed therapies will likely continue to reinforce a default preference for UFH, even in patients better suited for LMWH.

The trends in oral anticoagulation observed in this study align with prior research, with a decline in warfarin use in favor of DOACs, predominantly apixaban (31). Notably, roughly 55% of patients with high-risk PE received inpatient oral anticoagulation, compared to about 80% with non-high-risk PE. As current guidelines recommend at least three months of anticoagulation to prevent recurrence (6), transitioning to oral therapy before discharge is preferable. Adherence to subcutaneous LMWH is often lower due to cost, inconvenience, and the risk of painful hematomas or scarring (32). The lower inpatient use of oral anticoagulants in high-risk PE likely reflects a greater reliance on parenteral agents, with UFH or LMWH maintained throughout hospitalization, delaying the transition to oral therapy until after discharge.

### 4.3 Reperfusion therapies

Systemic thrombolysis remains the guideline-recommended first-line therapy for hemodynamic deterioration in high-risk and non-high-risk PE (6). In high-risk PE, where in-hospital mortality is estimated at 28.3% (33), rapid hemodynamic decline necessitates urgent intervention. Systemic thrombolysis is favored due to its ability to rapidly restore pulmonary perfusion and alleviate right ventricular strain, critical factors in preventing circulatory collapse (34). Additionally, in non-high-risk PE, current guidelines recommend systemic thrombolysis as the first-line rescue therapy for patients who experience hemodynamic deterioration while on anticoagulation (6). Although catheter-directed therapies are positioned as a potential alternative to systemic thrombolysis (14), their supporting evidence remains less robust, reinforcing systemic thrombolysis as the guideline-preferred approach (6, 28, 35).

Despite these recommendations, this study demonstrates a major shift in clinical practice, with CDE emerging as the predominant reperfusion strategy across non-high-risk and high-risk PE, findings consistent with prior studies (36, 37). Notably, the rapid acceleration in CDE utilization began in 2020, temporally coinciding with publication of the 2019 European Society of Cardiology PE guidelines (6), which more formally incorporated catheter-directed therapies into risk-based treatment algorithms. By 2024, approximately one in twelve non-high-risk patients and one in five high-risk patients received CDE.

These findings suggest that many centers now preferentially use CDE over systemic thrombolysis, a change likely driven by multiple factors. First, a growing proportion of patients with PE present with contraindications to systemic thrombolysis (38), making CDE an attractive alternative due to its ability to rapidly relieve pulmonary obstruction while mitigating the bleeding risk of systemic thrombolysis (39). Additionally, morbidity and mortality remain high despite existing management strategies, highlighting the need for innovation in PE treatment (40). Finally, the expanding institutional infrastructure for catheter-based PE management, particularly the proliferation of Pulmonary Embolism Response Teams (PERTs), has likely contributed to this shift. The implementation of PERTs is associated with increased utilization of advanced therapies, including catheter-directed therapies (41), reduced hospital length of stay, and lower mortality (42), making it an increasingly adopted model. As PERTs become more widely integrated, their multidisciplinary approach may further accelerate the transition toward catheter-based strategies in PE management.

Despite the growing adoption of CDE, comparative data remain limited (38). The recently completed PEERLESS trial (43), the first head-to-head randomized controlled trial (RCT) comparing interventional strategies for PE reperfusion, found that large-bore mechanical thrombectomy was associated with lower rates of clinical deterioration, bailout therapy, and postprocedural intensive care unit use when compared to catheter-directed thrombolysis in intermediate-risk PE. While these findings support the expanding role of CDE, they do not establish its efficacy relative to systemic thrombolysis, which remains the guideline-preferred approach for hemodynamic deterioration in PE.

The lack of large-scale RCTs directly comparing CDE with systemic thrombolysis highlights ongoing uncertainty regarding optimal patient selection and the appropriate role of CDE, particularly among patients without overt hemodynamic compromise. Moving forward, well-designed comparative trials are essential to define the role of CDE within guideline-directed care, ensuring its use is evidence-based and reserved for patients who require invasive therapy. Until such data emerge, continued evaluation of patient outcomes and careful refinement of patient selection criteria will be important to guide evidence-based integration of catheter-based interventions. Importantly, the appropriateness of specific reperfusion strategies cannot be determined using data from this study, as granular clinical severity measures, hemodynamics, imaging findings, and contraindications were not captured. Thus, these findings should be interpreted as descriptive of evolving practice patterns rather than as an assessment of guideline concordance or treatment appropriateness.

### 4.4 Hemodynamic support

This study demonstrates a growing preference for vasopressors and dobutamine in managing high-risk PE, with utilization increasing from 53.3% to 72.2% over the study period. Concurrently, the use of mechanical ventilation declined from 54.7% to 32.4%, reflecting another shift in clinical practice. Hemodynamic and respiratory support in PE remains complex and highly variable, with current guideline recommendations largely based on expert opinion, preclinical data, and observational studies (44). However, emerging evidence supports vasopressors as an initial approach for hemodynamic stabilization (45, 46), while mechanical ventilation has been increasingly associated with prolonged hospital stays, higher costs, and worse outcomes (44). As a result, there is growing consensus that mechanical ventilation should only be used when absolutely necessary, in favor of strategies that preserve spontaneous respiration and optimize right ventricular function.

### 4.5 COVID-19 pandemic considerations

Because this analysis spans 2016–2024, the COVID-19 pandemic likely influenced both PE epidemiology and inpatient management. Population-level data demonstrate a markedly increased short-term risk of PE following SARS-CoV-2 infection, particularly early in the pandemic and among patients with severe illness, contributing to a transient rise in thrombotic burden and hospitalized PE cases (47). Concurrently, early pandemic imaging patterns, characterized in some centers by fewer CT pulmonary angiograms but higher positivity, along with delayed presentations and strained healthcare systems may have shifted the hospitalized case mix toward more severe disease and worse short-term outcomes in patients with concurrent COVID-19 and PE (48). These factors may partially contextualize the rising proportion of high-risk PE observed during the study period. The pandemic also likely affected treatment selection: administrative analyses during COVID-era peaks describe shifts in advanced therapy utilization, including increased systemic thrombolysis and catheter-directed therapies and reduced use of resource-intensive surgical interventions, plausibly reflecting staffing limitations and procedural resource constraints (49). Accordingly, observed temporal trends in reperfusion strategies, ICU utilization, and supportive care in this study should be interpreted in the context of both secular evolution in PE management and pandemic-related perturbations rather than attributed to a single underlying cause.

### 4.6 Strengths and limitations

The large sample size and contemporary data make this study highly relevant, as it provides a comprehensive overview of current trends in PE management. Leveraging the Cosmos database, which reflects the population distribution of the U.S. Census, ensures broad generalizability. Physician-entered diagnoses, procedures, and medications enhance data accuracy, allowing for precise characterization of PE severity, treatment strategies, and inpatient management trends. Additionally, stratification by PE severity enables a risk-based evaluation of treatment patterns, offering insights that align with guideline-directed management.

However, this study has several important limitations. First, the Epic electronic health record system is costly to implement, therefore it is predominantly used by large academic and tertiary care centers, potentially overrepresenting hospitals with structured PE protocols, PERTs, and access to catheter-directed therapies. In contrast, smaller hospitals may rely more heavily on systemic thrombolysis for the acute management of hemodynamically unstable PE. Additionally, site-level identifiers are not available in the Cosmos database; therefore, hospital- or system-level clustering could not be explicitly accounted for. Together, these factors may lead to overestimation of adoption of catheter-directed therapies. Nevertheless, these findings highlight a rapidly growing preference for catheter-directed treatments, particularly CDE, underscoring their expanding role in PE management.

Because the unit of analysis was the hospitalization, inter-facility transfers may have resulted in multiple hospitalizations being counted for a single clinical PE episode, potentially modestly inflating hospitalization counts or procedural rates. Admission type (elective vs. non-elective) was not available; although PE was required to be the primary admitting diagnosis, a small number of planned procedural admissions may have been included. Furthermore, high-risk classification incorporated therapeutic interventions (mechanical ventilation, ECMO, and vasopressor or dobutamine use), which were also examined in descriptive trend analyses. Inclusion of these interventions in the exposure definition may introduce incorporation bias and inflate high-risk prevalence, particularly when evaluating temporal changes in advanced therapies. However, sensitivity analyses using a diagnosis-only definition of high-risk PE demonstrated directionally consistent trends across key outcomes.

Furthermore, high-risk cases were defined as those meeting any qualifying criterion at any point during hospitalization. While consistent with prior studies (22, 23), this approach may slightly overestimate the proportion of patients who present with high-risk PE, as it includes individuals who decompensate during their hospital stay despite initially stable presentations. In the PEITHO trial (50), which enrolled patients with intermediate-risk PE, hemodynamic decompensation or death occurred in 2.6% of patients treated with tenecteplase and 5.6% of those receiving placebo, highlighting that clinical deterioration, though uncommon, is not insignificant. Similarly, another study found that 4.1% of patients who were hemodynamically stable at presentation experienced in-hospital decompensation at 30 days (51). Taken together, these studies suggest that any overestimation of high-risk PE is likely limited to fewer than 5% of cases and does not materially alter the interpretation of our findings.

Finally, this study includes multiple comparisons across outcomes, time points, and risk strata. Formal adjustment for multiplicity was not performed, and subgroup findings should therefore be interpreted as descriptive and exploratory rather than confirmatory. These analyses were intended to characterize national practice patterns rather than to test prespecified mechanistic hypotheses.

## 5 Conclusion

This study provides a contemporary analysis of inpatient PE treatment trends in the U.S., highlighting the rapid adoption of CDE as the predominant reperfusion strategy. This substantial shift in practice underscores the need for continued comparative evidence to clarify the optimal role of CDE among available reperfusion approaches. The growing reliance on UFH, DOACs, and vasopressors further reflects evolving treatment patterns. These findings offer critical insights into national practice patterns and trends, with important implications for clinical decision-making and future guideline development.

## Data Availability

The datasets presented in this article are not readily available because direct access to the database is available only to individuals affiliated with, and approved by, a participating Cosmos healthcare organization. Requests to access the datasets should be directed to https://cosmos.epic.com/request-access/.
