# Supplementary material for: Contemporary national trends in the management of admissions for pulmonary embolism
Source: Front Cardiovasc Med. 2026 Mar 13;13:1757697. doi: 10.3389/fcvm.2026.1757697 (PMC13021881; doi:10.3389/fcvm.2026.1757697)
Supplement: Supplementary file 1 [file Datasheet1.docx]

**Supplemental Table 1.** ICD-10-CM, ICD-10-PCS, and CPT codes used to define high-risk criteria

| **High-risk criteria** | **Code** | **Description** |
| --- | --- | --- |
| Cardiac arrest | I46 | Cardiac arrest |
| Cardiogenic shock | R57.0 | Cardiogenic shock |
|  | R57.9 | Shock, unspecified |
| Mechanical ventilation | 5A1935Z | Respiratory Ventilation, Less than 24 Consecutive Hours |
|  | 5A1945Z | Respiratory Ventilation, 24-96 Consecutive Hours |
|  | 5A1955Z | Respiratory Ventilation, Greater than 96 Consecutive Hours |
| ECMO | 33946 | ECMO/extracorporeal life support (ECLS) provided by physician; initiation, veno-venous |
|  | 33947 | ECMO/ECLS provided by physician; initiation, veno-arterial |
|  | 33948 | ECMO/ECLS provided by physician; daily management, each day, veno-venous |
|  | 33949 | ECMO/ECLS provided by physician; daily management, each day, veno-arterial |
|  | 33952 | ECMO/ECLS provided by physician; insertion of peripheral (arterial and/or venous) cannula(e), percutaneous, 6 years and older |
|  | 33954 | ECMO/ECLS provided by physician; insertion of peripheral (arterial and/or venous) cannula(e), open, 6 years and older |
|  | 33956 | ECMO/ECLS provided by physician; insertion of central cannula(e) by sternotomy or thoracotomy, 6 years and older |
|  | 33958 | ECMO/ECLS provided by physician; reposition peripheral (arterial and/or venous) cannula(e), percutaneous, 6 years and older |
|  | 33962 | ECMO/ECLS provided by physician; reposition peripheral (arterial and/or venous) cannula(e), open, 6 years and older |
|  | 33964 | ECMO/ECLS provided by physician; reposition central cannula(e) by sternotomy or thoracotomy, 6 years and older |
|  | 33966 | ECMO/ECLS provided by physician; removal of peripheral (arterial and/or venous) cannula(e), percutaneous, 6 years and older |
|  | 33984 | ECMO/ECLS provided by physician; removal of peripheral (arterial and/or venous) cannula(e), open, 6 years and older |
|  | 33986 | ECMO/ECLS provided by physician; removal of central cannula(e) by sternotomy or thoracotomy, 6 years and older |
| Vasopressors/dobutamine | 3E030XZ | Introduction of Vasopressor into Peripheral Vein, Open Approach |
|  | 3E033XZ | Introduction of Vasopressor into Peripheral Vein, Percutaneous Approach |
|  | 3E040XZ | Introduction of Vasopressor into Central Vein, Open Approach |
|  | 3E043XZ | Introduction of Vasopressor into Central Vein, Percutaneous Approach |
|  | Other | Hospital or clinic administered norepinephrine or dobutamine |

**Supplemental Table 2.** ICD-10-CM codes used to define comorbid conditions

| **Comorbidity** | **Code** | **Description** |
| --- | --- | --- |
| Anemia | D50 | Iron deficiency anemia |
|  | D51 | Vitamin B12 deficiency anemia |
|  | D52 | Folate deficiency anemia |
|  | D53 | Other nutritional anemias |
|  | D55 | Anemia due to enzymatic disorders |
|  | D56 | Thalassemia |
|  | D57 | Sickle-cell disorders |
|  | D58 | Other hereditary hemolytic anemias |
|  | D59 | Acquired hemolytic anemia |
|  | D60 | Acquired pure red cell aplasia |
|  | D61 | Other aplastic anemias and other bone marrow failure |
|  | D62 | Acute posthemorrhagic anemia |
|  | D63 | Anemia in chronic diseases classified elsewhere |
|  | D64 | Other anemias |
| Cancer | C00-C96 | Malignant neoplasms |
| Coronary artery disease | I20 | Angina pectoris |
|  | I25 | Chronic ischemic heart disease |
| Diabetes mellitus | E10 | Type 1 diabetes mellitus |
|  | E11 | Type 2 diabetes mellitus |
|  | E12 | Other specified diabetes mellitus |
| Dyslipidemia | E78 | Disorders of lipoprotein metabolism and other lipidaemias |
| Heart failure | I50 | Heart failure |
| Hypertension | I10 | Essential (primary) hypertension |
|  | I11 | Hypertensive heart disease |
|  | I12 | Hypertensive chronic kidney disease |
|  | I13 | Hypertensive heart and chronic kidney disease |
|  | I15 | Secondary hypertension |

**Supplemental Table 3.** ICD-10-PCS codes used to define reperfusion procedures

| **Procedure** | **Code** | **Description** |
| --- | --- | --- |
| Catheter-directed embolectomy | 02CP3ZZ | Extirpation of Matter from Pulmonary Trunk, Percutaneous Approach |
|  | 02CQ3ZZ | Extirpation of Matter from Right Pulmonary Artery, Percutaneous Approach |
|  | 02CR3ZZ | Extirpation of Matter from Left Pulmonary Artery, Percutaneous Approach |
| Catheter-directed thrombolysis | 3E06317 | Introduction of Other Thrombolytic into Central Artery, Percutaneous Approach |
| Surgical embolectomy | 02CP0ZZ | Extirpation of Matter from Pulmonary Trunk, Open Approach |
|  | 02CQ0ZZ | Extirpation of Matter from Right Pulmonary Artery, Open Approach |
|  | 02CR0ZZ | Extirpation of Matter from Left Pulmonary Artery, Open Approach |
| Systemic thrombolysis | 3E03317 | Introduction of Other Thrombolytic into Peripheral Vein, Percutaneous Approach |

**Supplemental Table 4.** Wilson score 95% confidence intervals (CI) for surgical embolectomy and extracorporeal membrane oxygenation

|  | **Non-high-risk** | **High-risk** | |
| --- | --- | --- | --- |
| **Year** | **Surgical embolectomy**  **% (95% CI)** | **Surgical embolectomy**  **% (95% CI)** | **Extracorporeal membrane oxygenation**  **% (95% CI)** |
| 2016 | 0.08 (0.03-0.19) | 3.99 (2.24-6.99) | 3.62 (1.98-6.54) |
| 2017 | 0.09 (0.05-0.17) | 4.46 (2.97-6.66) | 2.23 (1.25-3.95) |
| 2018 | 0.07 (0.04-0.12) | 2.25 (1.41-3.58) | 4.77 (3.47-6.54) |
| 2019 | 0.05 (0.03-0.10) | 2.35 (1.54-3.56) | 3.91 (2.83-5.40) |
| 2020 | 0.06 (0.03-0.10) | 1.99 (1.29-3.05) | 1.99 (1.29-3.05) |
| 2021 | 0.06 (0.04-0.09) | 1.36 (0.93-1.99) | 2.04 (1.50-2.78) |
| 2022 | 0.04 (0.02-0.06) | 1.22 (0.87-1.72) | 1.91 (1.45-2.51) |
| 2023 | 0.05 (0.03-0.07) | 1.18 (0.87-1.61) | 2.75 (2.25-3.36) |
| 2024 | 0.04 (0.02-0.05) | 0.49 (0.32-0.77) | 2.06 (1.65-2.55) |

**Supplemental Figure 1.** Sensitivity analysis of trends in the proportion of admissions for high-risk pulmonary embolism using diagnosis-defined high-risk criteria

**Supplemental Figure 2.** Sensitivity analysis of parenteral anticoagulation use among diagnosis-defined high-risk pulmonary embolism admissions

LMWH = low-molecular-weight heparin; UFH = unfractionated heparin.

**Supplemental Figure 3.** Sensitivity analysis of oral anticoagulation use among diagnosis-defined high-risk pulmonary embolism admissions

**Supplemental Figure 4.** Sensitivity analysis of reperfusion therapy utilization among diagnosis-defined high-risk pulmonary embolism admissions

CDE = catheter-directed embolectomy; CDT = catheter-directed thrombolysis; SE = surgical embolectomy; ST = systemic thrombolysis.

**Supplemental Figure 5.** Sensitivity analysis of cardiopulmonary support among diagnosis-defined high-risk pulmonary embolism admissions

ECMO = extracorporeal membrane oxygenation.
